# Global gene expression analysis provides insight into local adaptation to geothermal streams in tadpoles of the Andean toad *Rhinella spinulosa*

**DOI:** 10.1038/s41598-017-01982-z

**Published:** 2017-05-16

**Authors:** Luis Pastenes, Camilo Valdivieso, Alex Di Genova, Dante Travisany, Andrew Hart, Martín Montecino, Ariel Orellana, Mauricio Gonzalez, Rodrigo A. Gutiérrez, Miguel L. Allende, Alejandro Maass, Marco A. Méndez

**Affiliations:** 1 Center for Genome Regulation, Blanco Encalada 2085, Santiago, Chile; 2 Departamento de Biología y Química, Facultad de Ciencias Básicas, Universidad Católica del Maule, Av. San Miguel 3605, Talca, Chile; 3 Laboratorio de Genética y Evolución, Departamento de Ciencias Ecológicas, Facultad de Ciencias, Universidad de Chile, Las Palmeras 3425, Santiago, Chile; 4 Center for Mathematical Modeling, Universidad de Chile, Beauchef 851, Santiago, Chile; 5 Centro de Investigaciones Biomédicas, Universidad Andrés Bello, República 239, Santiago, Chile; 6 Instituto de Nutrición y Tecnología de los Alimentos, Universidad de Chile, El Líbano 5524, Santiago, Chile; 7 Departamento de Biología, Facultad de Ciencias, Universidad de Chile, Las Palmeras 3425, Santiago, Chile; 8 Departamento de Ingeniería Matemática, Facultad de Ciencias Físicas y Matemáticas, Universidad de Chile, Beauchef 851, Santiago, Chile; 9 Instituto de Ecología y Biodiversidad, Departamento de Ciencias Ecológicas, Facultad de Ciencias, Universidad de Chile, Las Palmeras 3425, Santiago, Chile

## Abstract

The anuran *Rhinella spinulosa* is distributed along the Andes Range at altitudes that undergo wide daily and seasonal variation in temperature. One of the populations inhabits geothermal streams, a stable environment that influences life history traits such as the timing of metamorphosis. To investigate whether this population has undergone local adaptation to this unique habitat, we carried out transcriptome analyses in animals from two localities in two developmental stages (prometamorphic and metamorphic) and exposed them to two temperatures (20 and 25 °C). RNA-Seq, *de novo* assembly and annotation defined a transcriptome revealing 194,469 high quality SNPs, with 1,507 genes under positive selection. Comparisons among the experimental conditions yielded 1,593 differentially expressed genes. A bioinformatics search for candidates revealed a total of 70 genes that are highly likely to be implicated in the adaptive response of the population living in a stable environment, compared to those living in an environment with variable temperatures. Most importantly, the population inhabiting the geothermal environment showed decreased transcriptional plasticity and reduced genetic variation compared to its counterpart from the non-stable environment. This analysis will help to advance the understanding of the molecular mechanisms that account for the local adaptation to geothermal streams in anurans.

## Introduction

Local adaptation is the genetic change that occurs in a population in response to a localized geographical selective pressure^1^. It has been shown that in ectotherms temperature can be a critical factor during local adaptation^2^, which is expressed as differences in physiological, morphological and life history traits between populations that inhabit thermally contrasting environments in fishes^3, 4^, amphibians^5–7^ and reptiles^8, 9^. In the case of amphibians, it has been shown that temperature is the most important environmental variable that influences the expression of ecological requirements, physiological traits and also larval development^10–12^. The last is due to the strong dependence of the processes of growth and morphological differentiation on the thermal condition of the environment^13–15^. Although there are numerous studies about differentiation in these attributes, only a few have examined the genetic bases of this variation in amphibians^6, 16–18^.

*Rhinella spinulosa* Wiegmann, 1834, is a bufonid anuran that has a wide geographic distribution in South America, ranging from the Peruvian-Bolivian Altiplano to the eastern and western slopes of the Andes in Chile and Argentina^19^. This species is found in Chile from 17°30′ to 41°30′ S latitude and 1,400 to 4,580 m altitude^20^. *R. spinulosa* inhabits heterogeneous environments and presents genetic differentiation^21, 22^, as well as differences in morphological and life history traits^23–25^ among populations. Water temperature is one of the factors that has been reported as a modulator of the physiological, morphological and life history traits of larvae of *R. spinulosa* ^26^. Méndez & Correa-Solis^23^ studied two populations of *R. spinulosa* (El Tatio and Farellones, Chile) that inhabit different environmental temperature regimes. Larvae from El Tatio live in streams with a geothermal origin where water remains at a constant temperature of 25 ± 1.3 °C, as opposed to larvae from Farellones which live in variable water temperatures (4–30 °C). They found that when larvae from El Tatio were grown in the laboratory conditions at 20 °C there was a delay in early differentiation, increased stage-specific size, extended developmental time and higher mortality rate compared to individuals grown at 25 °C and to individuals from Farellones grown at either temperature. Based on these results, the existence of local adaptation to water temperature among individuals that inhabit the geothermal streams of El Tatio was proposed^23^. Nevertheless, a common garden experiment performed with larvae from a third Chilean population, Catarpe, where the same thermal treatment (20 and 25 °C) was evaluated, showed a higher survival rate at both temperatures than that reported for Farellones and El Tatio. In addition, tadpoles from Catarpe showed greater age at metamorphosis at 25 °C compared to the other two populations, while the growth rate at 25 °C was not higher than that observed for the postmetamorphics from El Tatio and Farellones. Considering that effects of biological interference (aquatic invertebrates and/or terrestrial predators) have not been reported in these populations, we propose that the pattern of variation in morphological and life history traits observed could be attributed to local adaptation to temperature.

**Figure 1:**
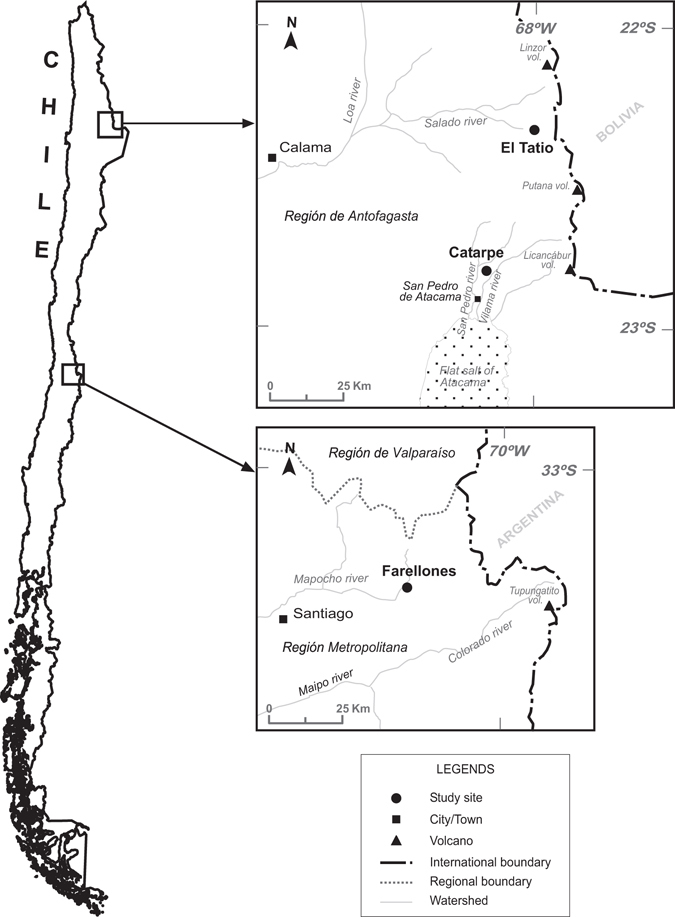
Sampling sites where egg collection of *Rhinella spinulosa* was carried out. Región de Antofagasta, Chile (top): El Tatio Geysers, geothermal permanent streams, and Catarpe valley, permanent streams with a daily thermal gradient. Región Metropolitana, Chile (bottom): mountain plains near Farellones, the control locality. The map was generated from digital information available at Google Earth Pro v7.1.4.1529 (https://www.google.com/intl/es/earth/) modified with FreeHand MX v11.0.2 software.

While variation in populations of *R. spinulosa* has been documented as a function of geographic origin for genetic, morphological and life history traits, an explanation of the regulatory mechanisms accounting for the differences detected in size and age at metamorphosis has not been proposed^21–25^. In this study we use RNA-Seq to examine the differential transcriptomic response that mediates local adaptation to temperature in *R. spinulosa*. The data presented here are the result of RNA-Seq analyses of tadpoles grown in a common garden experiment at two temperature regimes, 20 and 25 °C. The aim of this experiment was to compare the transcriptomic responses of tadpoles living in habitats that present thermal differences, El Tatio and Catarpe (Fig. 1). The geysers of El Tatio (4,260 m elevation), located 97 km NE of the town of San Pedro de Atacama, Región de Antofagasta, are a system of multiple geothermal streams in which amphibian larvae inhabit places where water temperature reaches 25 ± 1.3 °C, remaining constant throughout the whole year. In contrast, Catarpe (2,460 m), located 8 km NW of San Pedro de Atacama and 98 km SW of El Tatio, is a locality that shows thermal water variation (around 24 °C at noon and 10 °C at midnight).

The gene expression differences, combined with the geographic distance among the studied localities, provide a remarkable model to study local adaptive response associated with water temperature variation.

## Results

### RNA-Seq experiment and *de novo* assembly

Full-sib clutches from the two localities under study (Fig. 1) and a control locality (Farellones, Región Metropolitana, see Methods) were collected and raised under controlled conditions in a common garden experiment. Two temperatures (20 and 25 °C) and two developmental stages (prometamorphic and metamorphic) were used, for a total of 12 samples for each replicate. RNA was extracted and sequenced, generating a total of 1,761,618,602 reads of 101 bp in length, which represents 177.9 Gb with an average GC content of 47.2%. After the filtering process, the total reads were reduced to 1,729,315,143 (98.2%). The details of the sequencing results are provided in Tables S1, S2 and S3. As the reads represent only small fragments of RNA, we initially tried to align reads to available reference amphibian genomes (*Xenopus laevis* and *X. tropicalis*). However, only 5% of the reads produced a reliable match with these genomes, indicating significant sequence divergence between these two genera (*Rhinella* and *Xenopus*). The differences found at the nucleotide level between these anurans are likely due to the long evolutionary divergence time between them (230 million years approximately^27^). We then performed a *de novo* assembly using reads from replicate 1 to generate a reference transcriptome for *R. spinulosa*. The assembly had a total of 65,762 transcripts, a mean length of 1,252 bases and a peak of insert sizes between 300 and 400 bases. Using the assembled *R. spinulosa* transcriptome, approximately 31.9% of the transcripts (20,947) showed matches with known genes in the available databases, similar to the results of Yang *et al*.^28^ for the anurans *Rana chensinensis* and *R. kukunoris*.

After mapping the reads back to the reference transcriptome (79.1% of filtered reads on average), a matrix that assigns a raw expression value to each transcript under each experimental condition was built. With this matrix we built a boxplot to visualize the counts per million (CPM) of reads distributions among localities, thermal treatments and developmental stages. The boxplot showed a consistent distribution of CPM (Figure S1).

### SNP discovery and genetic variation

**Figure 2:**
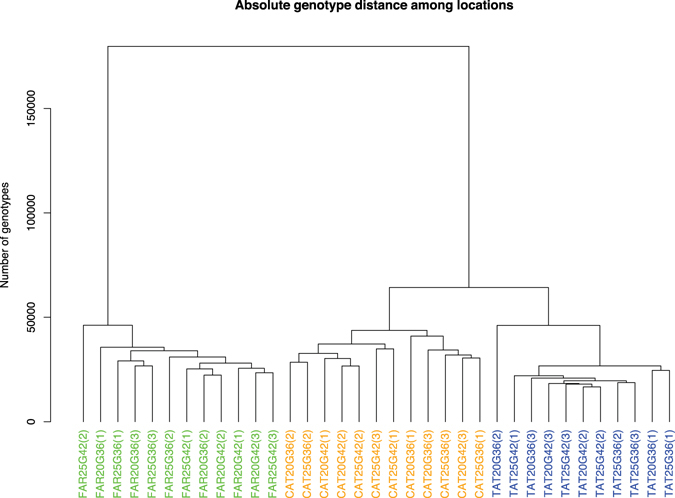
A dendrogram based on 194,469 SNPs showing the genotypic variation among the 35 samples of *Rhinella spinulosa*.

A search for SNP variants was performed with the resulting transcriptomes. We determined 194,469 high quality SNPs, which were used to construct a dendrogram of absolute genetic distance for the 12 experimental conditions and their respective replicates (Fig. 2). The dendrogram revealed that experimental conditions were grouped by locality; Farellones (control locality) exhibited a large genetic distance with respect to El Tatio and Catarpe; the latter localities had a small genetic distance. Then we computed pairwise genetic distances among localities using the F_ST_ index. The F_ST_ index value for El Tatio and Catarpe was 0.20207, for Catarpe and Farellones 0.47576, and for El Tatio and Farellones 0.54778. Due to the F_ST_ index values estimated for Farellones and to the marked genetic differences shown by the SNP analysis (Fig. 2), this population was excluded from the screening of candidate genes for temperature adaptation.

Finally, a dN/dS analysis was performed to identify genes under positive selection (PS) in the samples from El Tatio and Catarpe. The result revealed 488 genes in El Tatio and 1,018 in Catarpe (Tables S6 and S7).

### Genes differentially expressed in response to temperature

We identified those genes whose expression levels varied significantly only in response to temperature for each locality under the log-linear model described in Methods. For the two localities we found a total of 1,593 differentially expressed (DE) genes. Of these DE genes, 186 (11.7%) corresponded to El Tatio (Table S4) and 1,407 (88.3%) to Catarpe (Table S5). These two localities shared a total of 127 DE genes. Moreover, 59 (31.7%) DE genes were unique to El Tatio and 1,280 (90.9%) are unique to Catarpe.

**Figure 3:**
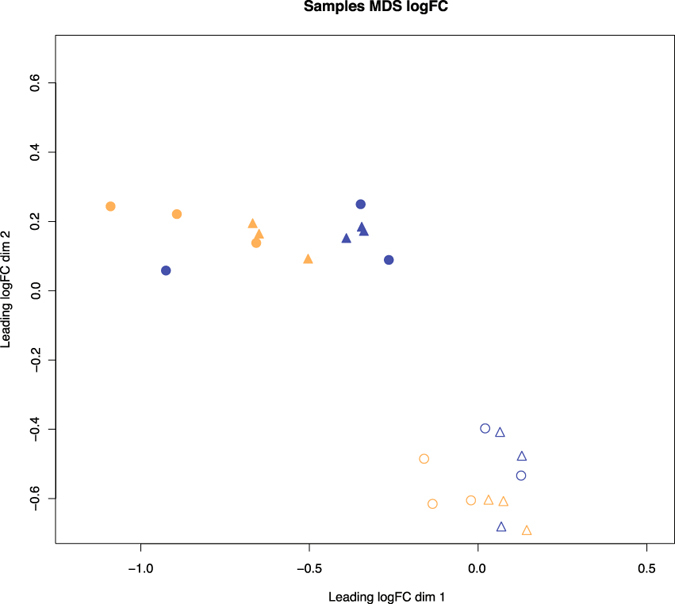
Multidimensional scaling (MDS) plot of the logarithm of fold-change (logFC) values for the most variable genes in the 35 assembled transcriptomes of *Rhinella spinulosa*. Symbols for El Tatio, Chile are shown in blue and Catarpe, Chile in orange. Circles correspond to treatment at 20 °C, and triangles to treatment at 25 °C; while the filled circles/triangles correspond to Gosner stage 36 and open circles/triangles to Gosner stage 42.

We performed a multi-dimensional scaling (MDS) plot, comparing samples based on log fold-change in expression level of the DE genes (Fig. 3). Gene expression variation was primarily explained by developmental stage and by locality, but less so by temperature. Furthermore, the populations from El Tatio and Catarpe were close to each other in the multidimensional space. This result concords with the dendrogram based on the SNPs shown above (Fig. 2).

A gene enrichment analysis for biological processes of these DE genes in response to temperature found enrichment in 193 categories in Catarpe (for details see Table S8), among which are “posttranscriptional regulation of gene expression” (GO:0010608), “regulation of translation elongation” (GO:0006448), “mRNA metabolic processes” (GO:0016071) and “SRP-dependent co-translational protein targeting to membrane” (GO:0006614)”. Additionally, no enriched categories were found in the sample from El Tatio.

Since the results showed marked differences between the transcriptomic responses of the individuals from the different localities studied, we decided to identify genes related to local adaptation by focusing on those genes that presented positive selection and that also displayed transcriptional variation. The results of combining these two selection criteria are summarized in Table 1.

**Table 1:** Summary of the results of the screening performed to find candidate genes for local adaptation to temperature in larvae of *Rhinella spinulosa* from El Tatio and Catarpe (Chile).

	El Tatio	Catarpe
T (°C), mean ± SE	25 ± 1.3	18 ± 2.2
DE genes	186	1,407
PS genes	488	1,018
Comparison*	2	68

DE, differentially expressed; PS, positive selection.

*Intersection between DE genes and PS genes.

### Identifying of candidate genes involved in local adaptation

SNP analysis did not show grouping based on developmental stage or thermal treatment. We therefore compared DE genes from the El Tatio population (stable environment) to those from Catarpe (variable environment). We found several *heat shock proteins* (*hsps*) that were differentially expressed in El Tatio and Catarpe. Four *hsps* showed differentiated expression in El Tatio (Table S4), all of which also displayed differentiated expression in Catarpe, but in the latter locality the total number of differentially expressed *hsps* was nine (Table S5).

As DE genes alone did not reveal a clear biological process that would discriminate between these two localities, we intersected DE genes with PS genes to add a second criterion for a deeper selection. Two genes were identified from the El Tatio population and 68 from Catarpe. The list of identified genes is shown in Table S9; these represent the most likely candidates that have a role in the adaptation of these populations of *R. spinulosa*. To examine this set of genes we searched for functional data associated with orthologs in GenBank, PubMed, Web of Science and Xenbase databases. Classification of these functions yielded cellular roles that include translation (ribosome biogenesis, rRNA synthesis and processing: 21.6%); gene regulation (transcription, chromatin modification, splicing: 20.6%); proteases and detoxification enzymes (12.2%); intracellular signalling (12.2%); extracellular matrix (9.5%) and cytoskeleton (8.1%). All of these functions can be associated with the major processes of embryogenesis and growth, consistent with the life stage under analysis. Further, we specifically searched for genes that have a function previously associated with temperature response in other species. We found no such genes in the gene set from El Tatio. However, in the examination of the set of genes from samples of Catarpe one gene satisfied this criterion: *transient receptor potential cation channel, subfamily V, member 2* (*trpv2*). This gene is interesting because it belongs to the thermoTRP superfamily and has been related to temperature homeostasis and thermal stress^29^, and was not differentially expressed in tadpoles from El Tatio.

Thus, our analysis of the data provided a set of candidate genes that are differentially expressed in response to temperature change in a common garden experimental setting and another set that are also under positive selection in a non-model organism, *Rhinella spinulosa*.

## Discussion

This study examines the transcriptomic differences between two thermal treatments (20 and 25 °C), at two specific developmental stages (prometamorphic and metamorphic) of *R. spinulosa*, obtained from two natural populations that inhabit distinct aquatic environments (El Tatio and Catarpe). In these environments, the most remarkable contrasting physical feature is the range of water temperature. The population that inhabits the geothermal streams from El Tatio has become locally adapted to a constant temperature regime (25 ± 1.3 °C), and performs poorly when exposed to lower temperatures under experimental conditions. Thus it was proposed that individuals from El Tatio have undergone a process of local adaptation to temperature^23^. How this adaptation occurred from the molecular-genetic point of view is unknown and focuses on the wider question of local adaptation as a mechanism of speciation or evolutionary divergence.

The transcriptomic data was first examined to assess the nucleotide variation present in the different populations sampled. Beyond the large volume of polymorphic sites found (194,469), cluster analysis reflected only a separation of the data by locality and not by stage of development or thermal treatment. Furthermore, the populations from El Tatio and Catarpe group together. The quantification of the genetic differentiation between these populations was supported by their F_ST_ values, where the comparison between El Tatio and Catarpe showed a moderate F_ST_ index (0.20207).

The dN/dS analysis allowed us to find genes under positive selection (PS) from the two populations studied. This type of analysis has been helpful, for example, to select candidate genes for adaptation to altitude using transcriptomes of amphibians^30^. The number of genes under PS in Catarpe in our study was twice the value obtained for the El Tatio population. However, the difference in the number of genes was not reflected in the F_ST_ value observed between these two populations. Also, the enrichment analysis for these genes under PS produced no common categories between the two populations or Gene Ontology terms specifically associated with temperature.

The analysis of DE genes was conducted using a log-linear model, which allowed us to recover genes whose differential expression was due only to temperature variability. This adjustment in the analysis allowed us to detect a total of 1,593 DE genes. Similar to what was observed in the dN/dS analysis, Catarpe showed a higher percentage of DE genes (88.3%). This is 8.6 times more DE genes than were found for El Tatio. This result is noteworthy because it reveals a heightened transcriptomic response in individuals of this population that compensates for the temperature change (from 25 to 20 °C). By comparing the DE gene lists we noted that El Tatio and Catarpe shared 127 DE genes, which correspond to 68.3% of the total DE genes of El Tatio. Interestingly, high transcriptomic plasticity was also recently reported in coral adaptation to a variable environment^31^.

A multivariate analysis for DE genes (MDS, Fig. 3) was also performed in order to examine the overall data under an independent perspective to that of the SNP calling. None of the groupings were explained by the temperature regimes; the enrichment by locality for these DE genes revealed no common GO terms between El Tatio and Catarpe. These results, together with the SNP evidence, led us to a deeper search for candidate genes by comparing the lists of PS and DE genes between the populations of El Tatio and Catarpe. This search yielded a total of 70 genes, 68 for Catarpe and 2 for El Tatio, representing a set of genes that are highly likely to be implicated in an adaptive response to temperature in this species (Table S9). Exploring the list of 70 genes for those related previously to temperature response, we were able to identify one (*trpv2*, from the Catarpe population list) that represents an attractive starting point for future functional analysis. The functional significance of this gene and its product in the adaptation to an environment where water temperature vary daily and seasonally, as they do in Catarpe, remains to be explored experimentally. In animals obtained from El Tatio geothermal streams, where water temperature is constant, this gene did not show any variation in expression under the experimental conditions tested.

Common garden experiments employing contrasting thermal treatments to assess different life history traits in postmetamorphics of *R. spinulosa* showed that the population from Catarpe was not affected by the change in temperature, unlike what was observed in El Tatio, where there was an increase in mortality and prolonged time to metamorphosis when individuals were exposed to 20 °C, a lower temperature than what larvae from this population experience in their natural geothermal stream habitat (25 ± 1.3 °C). The individuals from Catarpe showed more DE genes (88.3%), which suggests that this population exhibits an exacerbated and unstructured transcriptomic response (GO categories not related to temperature), but in which a few DE genes involved in this response have been associated with thermal stress (e.g., nine *heat shock proteins*). In contrast, El Tatio tadpoles presented a poor transcriptomic response (11.7% of DE genes), which correlates with the poor performance under thermal stress of individuals from this population. The *trpv2* gene that was differentially expressed and under positive selection in Catarpe, could thus be part of a compensatory mechanism to respond to stressful temperature changes. Furthermore, the lack of positive selection for this gene in the individuals from El Tatio could partially explain the low success in the survival of this population when larvae were exposed to a temperature lower than that experienced in their natural habitat.

We describe potential molecular players involved in the local adaptation to environmental temperature at a critical development stage of their life history for the first time in an ectothermic vertebrate. For further progress in understanding the mechanisms responsible for local adaptation to temperature it would be necessary to evaluate the identified candidate genes by quantifying their relative expression in individuals collected directly from natural populations along the entire distribution of *R. spinulosa*. Likewise, functional analyses through genetics and physiology, perhaps using laboratory models, can resolve whether the candidate genes detected in this study are in fact evolutionary targets of selection for adaptation to temperature.

## Conclusions

This study, carried out in wild populations of *Rhinella spinulosa* that inhabit contrasting thermal environments, suggests the existence of adaptive differential changes in gene expression related to temperature. Our findings indicate that in the case of individuals from El Tatio, the lack of differential expression of genes related to temperature, presumably as a consequence of living in a constant thermal environment, could be responsible for the prolonged time to metamorphosis and low survival rate reported for this population when these individuals are exposed to a temperature below that of their natural condition (25 ± 1.3 °C). In contrast, animals originating from a highly variable environment such as the Catarpe population display a wide transcriptional response to temperature change, indicative of greater plasticity and tolerance to variable environments. Candidate genes such as those proposed in this study could account for the genetic differentiation among populations that are under different selective environmental pressures. Furthermore, our study illustrates how a geothermal stream, a homogeneous and uncommon habitat for an amphibian, can shape the adaptive genetic mechanisms (e.g., lack of polymorphic variants) that become fixed in the population and how a variable environment such as Catarpe could modulate a broader transcriptomic response in larvae that live in this locality.

Finally, this study provides gene sequence data of a bufonid amphibian, a taxonomic group that includes more than 584 species in 50 genera, which are distributed worldwide except for the Antarctic continent^32^. *R. spinulosa* appears to be an excellent model in the family Bufonidae to study local adaptation, thus making possible a better understanding of the molecular bases and proximal mechanisms underlying this process.

## Methods

### Phenology and characteristics of the study sites

All the experimental procedures were carried out in accordance with the relevant guidelines and regulations approved by the Ethical Committee of the Faculty of Sciences of the Universidad de Chile.

The reproductive period of Chilean populations of *R. spinulosa* varies with altitude and latitude, and it is correlated with temperature and precipitation^33^. We selected populations (Fig. 1) that had a historical record of the physicochemical characteristics of their habitats and their phenology. In the geysers of El Tatio, Región de Antofagasta (22°20′10″S, 68°00′59″W), reproduction occurs throughout the whole year in geothermal streams, sites where the temperature remains constant (25 ± 1.3 °C). In the locality of Catarpe, Región de Antofagasta (22°50′02″S, 68°11′55″W), reproduction occurs between May and September in permanent ponds, which report thermal variation. A summary of information on several physicochemical parameters of water for the sampling sites is shown in Table 2. In addition, the population of Farellones, Región Metropolitana (33°21′02″S, 70°18′59″W, elevation 2,330 m) was considered as a control population due to its genetic differentiation^21^ and geographic distance (more than 1,700 km) from El Tatio and Catarpe.

**Table 2:** Altitude and three physicochemical parameters of the water are indicated for the localities studied (El Tatio and Catarpe, Chile) and the common garden experiment.

Parameters	Sampled localities		Common garden
	El Tatio	Catarpe	
Altitude (m)	4,260	2,460	585
T (°C)	25 ± 1.3	18 ± 2.2	20 ± 0.5 or 25 ± 0.5
pH	7.9 ± 0.0	7.8 ± 0.1	8.3 ± 0.02
Oxygen (mg/L)	5 ± 0.4	3.2 ± 0.1	8.1 ± 0.03

The values are shown as mean ± standard error, except for altitude.

Between October 2010 and August 2011, three wild full-sib clutches at (15–19) interval developmental Gosner stage were collected from each study site. We checked *in situ* that each clutch collected corresponded to full-sibs, as each clutch is a single cord of eggs having a beginning and an end. Although multiple paternity has not been observed in *R. spinulosa*, we cannot rule out the presence of this phenomenon in our study, given the regularity of multiple paternity in several other amphibian species^34–37^. All clutches were transported separately to the Laboratorio de Genética y Evolución (Facultad de Ciencias, Universidad de Chile, Santiago, Chile) for further analysis.

### Common garden experiment

A common garden experiment was performed in a climate chamber with a 12/12 L/D photoperiod. The collected eggs of *R. spinulosa* were placed in water tanks (2 L) at 20 °C until the larvae hatched and reached Gosner stage 25^38^. Larvae (full-sibs) at this stage were placed individually into 200 ml volume containers, with 25 replicates for each family (two families in total). Experimental temperature treatments were generated using submersible aquarium heaters in two separate containers (20 °C and 25 °C). Tadpoles were fed with boiled lettuce *ad libitum* and water was changed twice weekly. The diet was selected based on the report of Benavides *et al*.^39^, who did not find differences in assimilation efficiency of tadpoles of different geographical origin. Values of pH and oxygen concentration of the water were kept similar to those recorded at the sampling sites (Table 2).

We employed an experimental design of full-sibs from three localities: El Tatio, Catarpe and Farellones (experimental control). Two thermal treatments were used: 20 and 25 °C. These temperatures were selected because: (i) The individuals from El Tatio, which show local adaptation to temperature in stable geothermal streams (25 ± 1.3 °C), exhibit differences in their morphological and life history traits when exposed continuously to 20 °C^23^; and (ii) according to water temperature records from each locality (Table 2), the thermal treatment at 20 °C was selected considering an extensive field study on life history and larval ecology of 19 populations of *R. spinulosa* (2000–2015), where the average water temperature recorded was approximately 20 °C (19.2 °C; personal records by Marco A. Méndez). In addition, the Catarpe population is not genetically differentiated from El Tatio^22^.

We sampled whole individuals for each experimental treatment at Gosner stages 36 (prometamorphic) and 42 (metamorphic). In summary, the experiment comprises three replicates (the full-sib groups) for each of 12 experimental conditions (3 localities × 2 thermal treatments × 2 developmental stages).

All individuals were sacrificed by immersion in a solution of 0.2% (p/v) tricaine metansulfonate (Veterquímica Ltda., Chile). Samples were preserved independently in RNA*later* solution (Ambion Inc., Austin, TX) and stored at −20 °C.

### RNA extraction

RNA was isolated from sampled individuals of *R. spinulosa* (40–70 mg of tissue per sample) for each experimental treatment. Total RNA was extracted using the RNeasy Mini Kit (QIAGEN, Valencia, CA), according to the manufacturer’s instructions. To avoid contamination by gDNA, samples were digested with DNase I (QIAGEN) during the extraction procedure. Finally, the quantity and quality of total RNA was examined with a NanoDrop^®^ ND-1000 spectrophotometer (NanoDrop products, Wilmington, DE), and its integrity evaluated by denaturing electrophoresis in 1% (p/v) agarose-formaldehyde gels.

Next, all total RNAs were standardized to 100 ng/ul. Equal sample volumes per experimental treatment were combined to produce twelve pools, thus each pool represented two full-sib individuals of one developmental state (Gosner stage 36 or 42), one thermal treatment (20 or 25 °C) and one locality (El Tatio, Catarpe or Farellones). These RNA pools were sent to Macrogen, Inc. (Seoul, South Korea) and were examined in an Agilent 2100 Bioanalyzer (Agilent Technologies Inc., Santa Clara, CA) before sequencing.

### Construction of cDNA libraries and Illumina sequencing

The mRNA present in each RNA pool was isolated and purified to construct ds-cDNA libraries using the reagents provided in the Illumina^®^ TruSeq^™^ RNA Sample Preparation Kit (Illumina Inc.). These ds-cDNA libraries were paired-end sequenced using the Illumina^®^ HiSeq^™^ 2000 platform (Illumina Inc., San Diego, CA). Three rounds of sequencing were performed to obtain biological replicates. Replicate 1 was sequenced by pooling 12 samples in two lanes. Replicates 2 and 3 were sequenced by pooling 24 samples in two lanes. For all samples we used reads of 101 bp and paired-end libraries with insert-size of 250 bp.

### Data filtering, *de novo* assembly and gene annotation

All reads generated were quality filtered using the FastX toolkit (http://hannonlab.cshl.edu/fastx_toolkit/). Reads shorter than 50 base pairs after the base quality filter (<Q20) were discarded. After filtering, reads from replicate 1 were assembled *de novo* using the Trinity assembler^40^. After assembling a reference transcriptome of *R. spinulosa*, we kept only the largest isoform per gene and reduced redundancy transcripts using CD-HIT^41^, obtaining 87,844 non-redundant transcripts. Each RNA-Seq sample was then aligned separately back to this consensus transcriptome using TopHat software^42^, with default parameters. Finally, we filtered transcripts having less than 50 reads aligned.

The functional annotation of the reference transcriptome was performed with a Blast search (*e-value* ≤ 1e-10)^43^ against the protein databases Uniprot, Swissprot, NR, TCDB, KEGG and PRIAM. A domain assignment was also performed using the InterPro software^44^ and the databases of protein domains. Gene names were assigned using the best BLASTx hits against *Xenopus tropicalis* using gene models from Ensembl database.

### SNP calling

SNP variants were called using the following strategy: First, PCR-duplicated reads were removed from each BAM file (TopHat alignments) using MarkDuplicates from the Picard tools suite (http://broadinstitute.github.io/picard) (version_1.138). Second, SNPs were called simultaneously in all samples using Samtools mpileup^45^ (version_0.1.9), excluding reads with a mapping quality lower than 20 and bases with a phred quality less than 13 (−q 20 and −Q 13, respectively). Only variants having a phred quality above 20 and at least 4 supporting reads were considered as raw. Third, a total of 700,367 raw SNPs were filtered by genotype quality (≥10), genotype missing rate (≥20% across 36 samples), minor allele frequency (≥0.01) and bi-allelic variants using VCFtools^46^ (version_0.1.12). A set of 194,469 SNPs passed all filters.

SNP effects on transcripts were predicted using the SNPeff program^47^ (version_LGPL3). Genes under positive selection within localities were determined using the dN/dS ratio by considering only genes having at least 5 variable SNPs within localities (MAF > 0.01). For each locality we defined a gene as positively selected if the dN/dS ratio was >1.

The mean genetic distances (F_ST_ index) were computed among localities using Weir and Cockerham’s estimator implemented in VCFtools and all pass-filter SNPs as input.

### Differentially expressed genes in response to temperature

Transcripts were quantified using raw counts of the number of pair reads that were successfully mapped to our reference transcriptome. Differentially expressed (DE) genes were then obtained with the aid of the EdgeR package for the R statistical software^48, 49^ (v3.10.2). The raw data comprised a 65,762 × 36 matrix of counts, where each column corresponds to a combination of localities, developmental stage, temperature and replicates (3 localities per 2 developmental stages per 2 temperature regimes per 3 replicates yields a total of 36 samples), and each row corresponds to a gene that was counted at least once in any sample. However, the first TAT20G42 replicate appears to be an outlier, being lumped within the cluster of Gosner stage 42 samples from Catarpe and El Tatio. Suspecting technical issues with the acquisition of this sample, it was excluded from all subsequent analyses.

Next, we applied a filter to remove those genes that failed to record at least 0.25 counts per million in 6 samples. This was done to ensure that each gene would be adequately represented in two experimental conditions. The filtering process left 42,288 genes for further analysis. Prior to filtering, 13.7% to 49.7% (with an average of 34.9%) of the gene counts in each sample were zero. After filtering, 2.7% to 14.2% (with an average of 6.6%) of the counts for each sample were zero. Furthermore, each of the 42,288 genes was counted at least 7 times in 6 of the 35 columns, as well as being counted a total of 86 times or more among all samples.

Library sizes were normalized to compensate for differences in RNA composition using the relative log expression method proposed by Anders & Huber^50^. To study the effect of temperature on gene expression levels we fitted a log-linear model with negative binomial errors using a nested factorial design consisting of an additive model including developmental stage and temperature effects (together with an intercept) within each locality:

1 $${\mathrm{log}{\rm{\mu }}}_{ijk}={l}_{i}+{d}_{ij}+{t}_{ik}$$

where *l*_*i*_ is the main effect for locality i, *d*_*ij*_ denotes the effect of developmental stage j within locality i and *t*_*ik*_ is the effect of temperature k within locality i. Index i ranges over values CAT (Catarpe) and TAT (El Tatio), while j is either 36 or 42 and k is either 20 or 25. With appropriate constraints on the model coefficients, this model allows the effect of temperature at each locality to be assessed while controlling any effect due to developmental stage.

### Enrichment analysis

The Gene Ontology (GO) tool^51^ was used for the analysis of expression data. The analysis of gene enrichment employed the lists of DE genes and genes under positive selection per locality with the GOrilla tool^52, 53^. A *p-value* ≤ 1 × 10^−3^ was used to consider a GO category as enriched. The GO enrichments were performed independently for the categories Biological Process, Cell Component and Molecular Function using as background the database of GO genes of *Homo sapiens*.

### Identification of candidate genes

A final set of candidate transcripts was obtained by combining positive selection (PS) data and differential expression (DE) results for the two genetically related populations, El Tatio and Catarpe. The annotated data for this set of transcripts was used to search for molecular or cellular functions associated with temperature response (GenBank, PubMed, Web of Science, and Xenbase databases).

## Electronic supplementary material

Table S4

Table S5

Table S6

Table S7

Table S8

Table S9

Supporting info

## References

[1] Kawecki TJ, Ebert D (2004). Conceptual issues in local adaptation. Ecol. Lett..

[2] Smith-Gill SJ, Berven KA (1979). Predicting amphibian metamorphosis. Am. Nat..

[3] Bergenius MAJ, Meekan MG, Robertson DR, McCormick MI (2002). Larval growth predicts the recruitment success of a coral reef fish. Oecologia..

[4] Grorud-Colvert K, Sponaugle S (2011). Variability in water temperature affects trait-mediated survival of a newly settled coral reef fish. Oecologia..

[5] Blouin MS, Brown ST (2000). Effects of temperature-induced variation in anuran larval growth rate on head width and leg length at metamorphosis. Oecologia..

[6] Laurila A, Karttunen S, Merilä J (2002). Adaptive phenotypic plasticity and genetics of larval life histories in two *Rana temporaria* populations. Evolution..

[7] Orizaola G, Dahl E, Nicieza A, Laurila A (2013). Larval life history and anti-predator strategies are affected by breeding phenology in an amphibian. Oecologia..

[8] Angilletta MJ, Niewiarowski PH, Dunham AE, Leaché AD, Porter WP (2004). Bergmann’s Clines in Ectotherms: Illustrating a Life-History Perspective with Sceloporine Lizards. Am. Nat..

[9] Terribile LC, Olalla-Tárraga MA, Diniz-Filho JAF, Rodríguez MA (2009). Ecological and evolutionary components of body size: geographic variation of venomous snakes at the global scale. Biol. J. Linnean Soc.

[10] Ultsch, G. R., Bradford, D. F. & Freda, J. Physiology: coping with the environment in *Tadpoles: The Biology of Anuran Larvae* (eds McDiarmid, R. W. & Altig, R.) 189–214 (The University of Chicago Press, Chicago 1999).

[11] Chen TC, Kam YC, Lin YS (2001). Thermal physiology and reproductive phenology of *Buergeria japonica* (Rhacophiridae) breeding in a stream and a geothermal hotspring in Taiwan. Zoolog. Sci..

[12] Olsson M, Uller T (2003). Thermal environment, survival and local adaptation in the common frog. Rana temporaria. Evol. Ecol. Res..

[13] Atkinson D (1994). Temperature and organism size – A biological law for ectotherms?. Adv. Ecol. Res.

[14] Álvarez D, Nicieza G (2002). Effects of temperature and food quality on anuran larval growth and metamorphosis. Func. Ecol..

[15] Tejedo M (2010). Contrasting effects of environmental factors during larval stage on morphological plasticity in post-metamorphic frogs. Clim. Res..

[16] Laugen AT, Laurila A, Merilä J (2002). Maternal and genetic contributions to geographical variation in *Rana temporaria* larval life-history traits. Biol. J. Linnean Soc..

[17] Laugen AT, Laurila A, Merilä J (2003). Latitudinal and temperature-dependent variation in embryonic development and growth in *Rana temporaria*. Oecologia..

[18] Laugen AT, Laurila A, Räsänen K, Merilä J (2003). Latitudinal countergradient variation in the common frog (*Rana temporaria*) developmental rates – evidence for local adaptation. J. Evol. Biol..

[19] IUCN SSC Amphibian Specialist Group. *Rhinella spinulosa. The IUCN Red List of Threatened Species: e.T54763A61394818* (2015). Available at: http://www.iucnredlist.org/details/54763/0. (Accessed: 7th November 2016).

[20] Lobos, G. *et al*. *Anfibios de Chile, un desafío para la conservación*. 99 (Ministerio del Medio Ambiente, Fundación Facultad de Ciencias Veterinarias y Pecuarias de la Universidad de Chile y Red Chilena de Herpetología. Santiago 2013).

[21] Méndez M (2004). Morphological and genetic differentiation among Chilean populations of *Bufo spinulosus* (Anura: Bufonidae). Rev. Chil. Hist. Nat..

[22] Correa C, Pastenes L, Sallaberry M, Veloso A, Méndez M (2010). Phylogeography of *Rhinella spinulosa* (Anura: Bufonidae) in northern Chile. Amphib-Reptil..

[23] Méndez MA, Correa-Solis M (2009). Divergence in morphometric and life history traits in two thermally contrasting Andean populations of *Rhinella spinulosa* (Anura: Bufonidae). J. Therm. Biol..

[24] Márquez-García M, Correa-Solis M, Sallaberry M, Méndez MA (2009). Effects of pond drying on morphological and life history traits in the anuran *Rhinella spinulosa* (Anura: Bufonidae). Evol. Ecol. Res..

[25] Márquez-García M, Correa-Solis M, Méndez MA (2010). Life-history trait variation in tadpoles of the warty toad in response to pond drying. J. Zool..

[26] Soto, E. R., Sallaberry, M., Núñez, J. J. & Méndez, M. A. Desarrollo larvario y estrategias reproductivas en anfibios in *Herpetología de Chile* (eds Vidal, M. & Labra, A.) 333–357 (Science-Verlag, Chile 2008).

[27] Roelants K (2007). Global patterns of diversification in the history of modern amphibians. P. Natl. Acad. Sci. USA.

[28] Yang W, Qi Y, Bi K, Fu J (2012). Toward understanding the genetic basis of adaptation to high-elevation life in poikilothermic species: A comparative transcriptomic analysis of two ranid frogs, *Rana chensinensis* and *R. kukunoris*. BMC Genomics..

[29] Saito S, Shingai R (2006). Evolution of thermoTRP ion channel homologs in vertebrates. Physiol. Genomics..

[30] Wang I (2012). Environmental and topographic variables shape genetic structure and effective population sizes in the endangered Yosemite toad. Divers. Distrib..

[31] Kenkel CD, Matz MV (2016). Gene expression plasticity as a mechanism of coral adaptation to a variable environment. Nat. Ecol. Evol.

[32] Frost, D. R. *Amphibian Species of the World: an Online Reference. Version 6.0*. American Museum of Natural History, New York, USA (2015). Available at: http://research.amnh.org/herpetology/amphibia/index.html. (Accessed: 7th November 2016).

[33] Veloso, A. *et al*. Contribución sistemática al conocimiento de la herpetofauna del extremo norte de Chile in *El hombre y los ecosistemas de montaña* 135–256 (MAB 6, Santiago, Chile 1982).

[34] Laurila A, Seppa P (1998). Multiple paternity in the common frog (*Rana temporaria*): genetic evidence from tadpole kin groups. Biol. J. Limn.Soc..

[35] Byrne PG, Roberts JD (2004). Intrasexual selection and group spawning in quacking frogs (*Crinia georgiana*). Behav. Ecol..

[36] Sztatesny M, Jehle R, Burke T, Hödl W (2006). Female polyandry under male harassment: the case of the common toad (*Bufo bufo*). J. Zool..

[37] Ayres C (2008). Multiple amplexus in the Iberian Brown Frog *Rana iberica. N. West*. J. Zool..

[38] Gosner KL (1960). A simplified table for staging anuran embryos and larvae with notes on identification. Herpetologica..

[39] Benavides AG, Veloso A, Jiménez P, Méndez MA (2005). Assimilation efficiency in *Bufo spinulosus* tadpoles (Anura: Bufonidae): effects of temperature, diet quality and geographic origin. Rev. Chil. Hist. Nat..

[40] Grabherr MG (2011). Full-length transcriptome assembly from RNA-seq data without a reference genome. Nature Biotechnol..

[41] Li W, Godzik A (2006). Cd-hit: a fast program for clustering and comparing large sets of protein or nucleotide sequences. Bioinformatics..

[42] Trapnell C, Pachter L, Salzberg SL (2009). TopHat: discovering splice junctions with RNA-Seq. Bioinformatics..

[43] Camacho C (2009). BLAST+: architecture and applications. BMC Bioinformatics..

[44] Hunter S (2012). InterPro in 2011: new developments in the family and domain prediction database. Nucleic Acids Res..

[45] Li H (2011). A statistical framework for SNP calling, mutation discovery, association mapping and population genetical parameter estimation from sequencing data. Bioinformatics..

[46] Danecek P (2011). The variant call format and VCFtools. Bioinformatics..

[47] Cingolani P (2012). A program for annotating and predicting the effects of single nucleotide polymorphisms, SnpEff: SNPs in the genome of *Drosophila melanogaster* strain w1118; iso-2; iso-3. Fly (Austin).

[48] Robinson MD, McCarthy DJ, Smyth GK (2010). edgeR: a Bioconductor package for differential expression analysis of digital gene expression data. Bioinformatics..

[49] McCarthy DJ, Chen Y, Smyth GK (2012). Differential expression analysis of multifactor RNA-Seq experiments with respect to biological variation. Nucleic Acids Res..

[50] Anders S, Huber W (2010). Differential expression analysis for sequence count data. Genome Biol..

[51] Ashburner M (2000). Gene Ontology: tool for the unification of biology. Nature Genet..

[52] Eden E, Lipson D, Yogev S, Yakhini Z (2007). Discovering Motifs in Ranked Lists of DNA Sequences. PLoS Comput. Biol..

[53] Eden E, Navon R, Steinfeld I, Lipson D, Yakhini Z (2009). GOrilla: A Tool for Discovery and Visualization of Enriched GO Terms in Ranked Gene Lists. BMC Bioinformatics..

