# Supplementary material for: Global gene expression analysis provides insight into local adaptation to geothermal streams in tadpoles of the Andean toad Rhinella spinulosa
Source: Sci Rep. 2017 May 16;7:1966. doi: 10.1038/s41598-017-01982-z (PMC5434060; doi:10.1038/s41598-017-01982-z)
Supplement: Supplementary file 7 — Supporting info [file 41598_2017_1982_MOESM7_ESM.pdf]

Global gene expression analysis provides insight into local adaptation to geothermal streams in tadpoles of the Andean toad *Rhinella spinulosa*

Luis Pastenes[1,2], Camilo Valdivieso[1,3], Alex Di Genova[1,4], Dante Travisany[1,4], Andrew Hart[4], Martín Montecino[1,5], Ariel Orellana[1], Mauricio Gonzalez[1,6], Rodrigo A. Gutiérrez[1], Miguel L. Allende[1,7], Alejandro Maass[1,4,8] & Marco A. Méndez[1,3,9,*]

SUPPLEMENTARY INFORMATION

Supplementary Tables:

Table S1. Summary of the results of the RNA sequencing and *de novo* assembled transcriptome for *Rhinella spinulosa*.

	**Final assembly**
Total number of reads (millions)	1,761.6
Read length (bp)	101
Total length of reads (Gb)	177.9
Total length of assembly (Mb)	82.3
Percentage sequences of assembly (%)	79.2
N50 length of assembly (bp)	1,936
Mean length of transcripts (bp)	1,252
Median length of transcripts (bp)	743
Total number of transcripts	65,762
Transcripts annotated	20,947

Table S2. Total number of reads resulting from the 36 cDNA libraries sequenced. The total number of reads is shown for each experimental condition, both before (total pairs) and after (pass filter) the trimming process. These include the singlets, forward and reverse reads.

Experimental conditions	Total pairs	Pass filter pairs	Pass filter singlets	Pass filter forward	Pass filter reverse
CAT20G36(1)	28,975,309	27,928,398	947,105	703,416	243,689
CAT20G36(2)	20,120,514	19,566,618	516,385	376,042	140,343
CAT20G36(3)	19,893,759	19,322,012	531,090	385,147	145,943
CAT20G42(1)	37,088,766	35,668,443	1,308,905	912,323	396,582
CAT20G42(2)	19,333,620	18,736,917	554,015	400,035	153,980
CAT20G42(3)	16,358,617	15,784,950	531,096	374,388	156,708
CAT25G36(1)	42,370,880	40,673,256	1,514,671	1,085,870	428,801
CAT25G36(2)	21,241,098	20,612,726	586,639	429,165	157,474
CAT25G36(3)	17,834,333	17,305,785	494,354	367,448	126,906
CAT25G42(1)	33,512,084	32,354,950	1,062,731	715,411	347,320
CAT25G42(2)	19,480,898	18,806,609	636,773	473,203	163,570
CAT25G42(3)	21,368,003	20,702,739	621,593	453,156	168,437
FAR20G36(1)	28,098,598	26,942,142	1,053,549	776,678	276,871
FAR20G36(2)	22,210,026	21,582,335	583,353	420,468	162,885
FAR20G36(3)	17,036,683	16,478,704	520,275	383,433	136,842
FAR20G42(1)	34,727,434	33,662,917	984,069	652,928	331,141
FAR20G42(2)	18,957,500	18,265,742	645,805	458,856	186,949
FAR20G42(3)	21,645,300	20,911,108	688,690	514,080	174,610
FAR25G36(1)	36,392,396	35,071,891	1,207,621	848,681	358,940
FAR25G36(2)	18,235,964	17,599,458	591,354	437,994	153,360
FAR25G36(3)	18,865,067	18,298,563	531,378	391,038	140,340
FAR25G42(1)	34,787,863	33,669,651	1,031,243	693,798	337,445
FAR25G42(2)	20,775,599	19,990,093	734,610	529,990	204,620
FAR25G42(3)	17,840,840	17,270,884	531,291	383,285	148,006
TAT20G36(1)	34,647,200	33,110,285	1,365,224	969,683	395,541
TAT20G36(2)	20,937,377	20,380,109	519,431	373,926	145,505
TAT20G36(3)	19,867,133	19,322,967	506,504	372,635	133,869
TAT20G42(1)	30,139,828	29,015,346	1,011,817	718,615	293,202
TAT20G42(2)	20,678,674	20,050,216	584,232	415,173	169,059
TAT20G42(3)	19,000,686	18,428,164	534,520	381,677	152,843
TAT25G36(1)	35,076,239	33,632,975	1,308,133	926,773	381,360
TAT25G36(2)	20,102,632	19,509,561	551,607	392,048	159,559
TAT25G36(3)	20,213,331	19,565,042	601,723	432,016	169,707
TAT25G42(1)	31,772,768	30,629,011	1,033,039	732,672	300,367
TAT25G42(2)	18,665,254	18,104,744	525,485	383,583	141,902
TAT25G42(3)	22,557,028	21,937,403	579,405	426,413	152,992
**Total**	880,809,301	850,892,714	27,529,715	19,692,047	7,837,668

Table S3. Total number of filtered and mapped reads for the 36 experimental conditions. These include forward, reverse and paired reads, as well as the overall read mapping and concordant pair alignment rates.

Experimental conditions	Total pairs	Mapped forward	Mapped reverse	Mapped pairs	Overall read mapping rate (%)	Concordant pair alignment rate (%)
CAT20G36(1)	27,928,398	22,515,045	22,436,706	20,412,351	80.5	72.4
CAT20G36(2)	19,566,618	15,808,607	15,826,055	15,031,787	80.8	76.7
CAT20G36(3)	19,322,012	15,512,062	15,523,688	14,678,529	80.3	75.9
CAT20G42(1)	35,668,443	28,462,682	28,382,262	25,709,558	79.7	70.9
CAT20G42(2)	18,736,917	15,035,048	15,056,284	14,280,474	80.3	76.1
CAT20G42(3)	15,784,950	12,871,018	12,891,571	12,256,130	81.6	77.5
CAT25G36(1)	40,673,256	32,490,346	32,355,852	29,024,159	79.7	70.5
CAT25G36(2)	20,612,726	16,405,499	16,427,714	15,561,201	79.6	75.4
CAT25G36(3)	17,305,785	13,996,733	14,017,691	13,329,757	80.9	76.9
CAT25G42(1)	32,354,950	25,703,894	25,660,876	23,250,623	79.4	70.8
CAT25G42(2)	18,806,609	15,025,113	15,054,349	14,259,766	80	75.7
CAT25G42(3)	20,702,739	16,524,163	16,552,595	15,688,818	79.9	75.7
FAR20G36(1)	26,942,142	20,610,947	20,529,417	17,838,563	76.4	65.3
FAR20G36(2)	21,582,335	16,215,973	16,252,426	15,018,572	75.2	69.5
FAR20G36(3)	16,478,704	12,418,830	12,450,435	11,384,633	75.5	69.0
FAR20G42(1)	33,662,917	25,345,993	25,344,617	22,196,234	75.3	65.4
FAR20G42(2)	18,265,742	13,861,159	13,905,631	12,816,129	76	70.1
FAR20G42(3)	20,911,108	15,629,513	15,671,596	14,460,096	74.8	69.1
FAR25G36(1)	35,071,891	26,514,025	26,463,804	23,042,816	75.5	64.9
FAR25G36(2)	17,599,458	13,274,447	13,314,862	12,247,760	75.5	69.5
FAR25G36(3)	18,298,563	13,837,279	13,875,838	12,840,499	75.7	70.1
FAR25G42(1)	33,669,651	25,612,349	25,597,513	22,452,622	76.1	66.0
FAR25G42(2)	19,990,093	15,400,623	15,446,449	14,318,102	77.2	71.6
FAR25G42(3)	17,270,884	13,047,526	13,086,089	12,096,674	75.7	69.9
TAT20G36(1)	33,110,285	26,707,607	26,626,774	24,068,936	80.5	71.5
TAT20G36(2)	20,380,109	16,717,665	16,732,698	15,949,366	82.1	78.2
TAT20G36(3)	19,322,967	15,669,693	15,683,865	14,845,980	81.1	76.7
TAT20G42(1)	29,015,346	23,375,848	23,325,455	20,977,018	80.5	71.5
TAT20G42(2)	20,050,216	16,304,895	16,330,244	15,513,854	81.4	77.2
TAT20G42(3)	18,428,164	14,919,046	14,941,834	14,204,358	81	76.9
TAT25G36(1)	33,632,975	27,963,540	27,884,801	25,732,973	83	75.8
TAT25G36(2)	19,509,561	15,975,422	15,994,754	15,242,394	81.9	78.0
TAT25G36(3)	19,565,042	16,133,812	16,152,313	15,383,554	82.5	78.5
TAT25G42(1)	30,629,011	24,569,964	24,520,998	22,407,288	80.1	72.4
TAT25G42(2)	18,104,744	14,718,491	14,740,339	14,020,157	81.4	77.3
TAT25G42(3)	21,937,403	18,068,868	18,087,377	17,192,798	82.4	78.3
**Total**	850,892,714	673,243,725	673,145,772	619,734,529	-	-

Table S4. List of differentially expressed genes from El Tatio. (Submitted as Excel file)

Table S5. List of differentially expressed genes from Catarpe. (Submitted as Excel file)

Table S6. List of genes with positive selection detected in the samples from El Tatio. The total number of SNPs and their effects are also shown. (Submitted as Excel file)

Table S7. List of genes with positive selection detected in the samples from Catarpe. The total number of SNPs and their effects are also shown. (Submitted as Excel file)

Table S8. Gene enrichment for the DE genes detected in the samples from Catarpe. (Submitted as Excel file)

Table S9. List of DE genes and with positive selection for the localities from El Tatio and Catarpe. (Submitted as Excel file)

Supplementary Figures (Legends):

Figure S1. Boxplot of the normalized library sizes (RLE method) for the 35 assembled transcriptomes of *Rhinella spinulosa*. The Y-axis shows the counts per million (CPM) mapped reads for each replicate of the twelve experimental conditions.

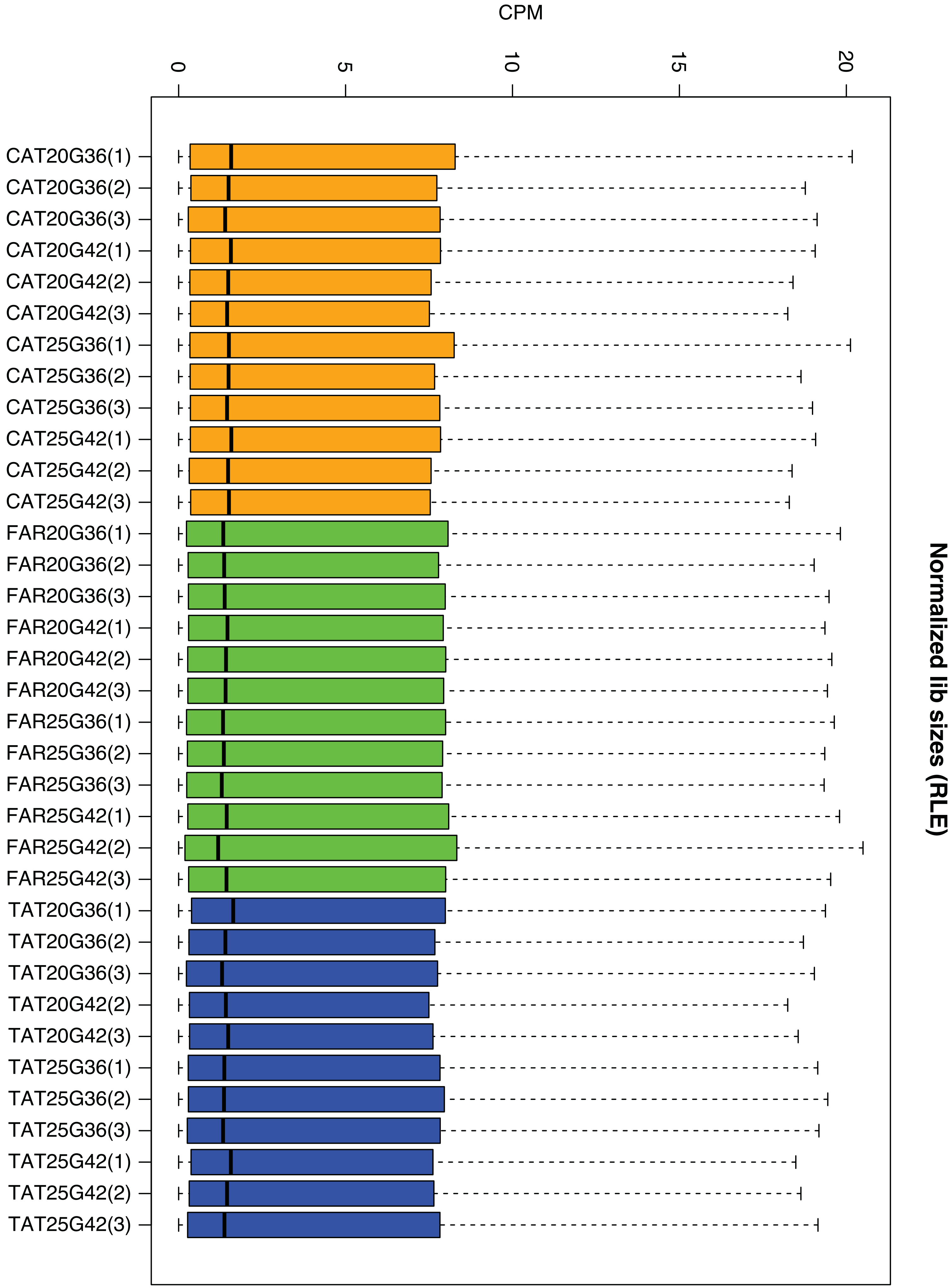

Normalized lib sizes (RLE)
CPM
0
5
10
15
20
CAT20G36(1)
CAT20G36(2)
CAT20G36(3)
CAT20G42(1)
CAT20G42(2)
CAT20G42(3)
CAT25G36(1)
CAT25G36(2)
CAT25G36(3)
CAT25G42(1)
CAT25G42(2)
CAT25G42(3)
FAR20G36(1)
FAR20G36(2)
FAR20G36(3)
FAR20G42(1)
FAR20G42(2)
FAR20G42(3)
FAR25G36(1)
FAR25G36(2)
FAR25G36(3)
FAR25G42(1)
FAR25G42(2)
FAR25G42(3)
TAT20G36(1)
TAT20G36(2)
TAT20G36(3)
TAT20G42(2)
TAT20G42(3)
TAT25G36(1)
TAT25G36(2)
TAT25G36(3)
TAT25G42(1)
TAT25G42(2)
TAT25G42(3)
